# Global distribution and coincidence of pollution, climate impacts, and health risk in the Anthropocene

**DOI:** 10.1371/journal.pone.0254060

**Published:** 2021-07-21

**Authors:** Richard Marcantonio, Debra Javeline, Sean Field, Agustin Fuentes

**Affiliations:** 1 The Joan B. Kroc Institute for International Peace Studies, University of Notre Dame, Notre Dame, Indiana, United States of America; 2 Department of Anthropology, University of Notre Dame, Notre Dame, Indiana, United States of America; 3 Department of Political Science, University of Notre Dame, Notre Dame, Indiana, United States of America; 4 Department of Anthropology, Princeton University, Princeton, New Jersey, United States of America; Shahjalal University of Science and Technology, BANGLADESH

## Abstract

Previous research demonstrates that low-income countries face higher risks than high-income countries from toxic pollution and climate change. However, the relationship between these two risks is little explored or tested, and efforts to address the risks are often independent and uncoordinated. We argue that the global risks from toxic pollution and climate change are highly correlated and should be jointly analyzed in order to inform and better target efforts to reduce or mitigate both risks. We provide such analysis for 176 countries and found a strong (r_s_ = -0.798;95%CI -0.852, -0.727) and significant (p<0.0001) relationship between the distribution of climate risk and toxic pollution. We also found that inequities in pollution production, economic status, and institutional readiness are interconnected and exacerbate risk for countries already in the highest risk categories for both toxic and non-toxic (greenhouse gas) pollution. The findings have policy implications, including the use of the proposed *Target* assessment to decide where best to address toxic and non-toxic pollution simultaneously, based on the need to minimize human suffering and maximize return on effort.

## Introduction

Human-produced pollution is destabilizing the entire Earth System [1–3]. This pollution poses severe risk to human health and the contemporary human niche [4–11]. Most research assesses the independent risks of toxic emissions (e.g. fine particulate matter or PM2.5) and non-toxic emissions (e.g. greenhouse gases) and people’s vulnerability to them [9,12,13]. However, these risks are intricately connected and substantially catalyze each other both in the environment and the human body [9–11,14–20]. For example, flood events disturb and redistribute toxic materials [16,21,22]. Research is lacking on the simultaneous and interactive risk from toxic and non-toxic emissions to human health [23]. There is a need for “bold and comprehensive strategies that simultaneously address both problems…to overcome the separation between pollution prevention and climate change mitigation” [23]. Numerous collaborative efforts are underway to rectify this shortfall—e.g., the new Center for Climate Change and the Center for Climate, Health and the Global Environment (C-CHANGE) at the Harvard TH Chan School of Public Health, the latter led until recently by Gina McCarthy, former Administrator of the US Environmental Protection Agency (2013–2017) and now the first White House National Climate Advisor [24]. However, no studies to date have tested the relationship between toxic pollution risk and climate risk to human health in order to inform these new efforts [7–11,17,23].

Such a gap in information and analyses restricts broadscale, and local, efforts to characterize specific patterns and processes of interaction accurately and holistically between toxic and non-toxic (climate impact) pollution. The relationships between the two processes, as compared to models that keep the two processes and corresponding risks separate, may be a better representation of the actual overall dynamic of challenges faced. Improved understanding of these interfaces offers institutions, agencies, and practitioners a greater toolkit with which to attempt amelioration of the harms resulting from pollution.

How does the human health risk due to toxic and non-toxic pollution vary by country? How strong is the relationship between the risk from each pollution type, and where specifically is the risk, and the potential to address the risk, greatest? To answer these questions, we join data measuring global climate risk and institutional capacity from the Notre Dame Global Adaptation Index (ND-GAIN) Country Index [12], environmental quality from the Yale Environmental Performance Index (EPI) [13], and toxic pollution-caused mortality from the Global Alliance on Health and Pollution (GAHP) [9], all for the year 2018, to explore the global distribution of human-produced or exacerbated environmental risks in multiple countries (N = 176). We hypothesize that there is a strong relationship between the global distribution of toxic pollution risk and climate impacts risk. Low- and middle-income countries are often unable to afford or enforce strict regulatory regimes against polluting industries or the infrastructure and other costs associated with reducing vulnerability to climate change and hazardous waste. Using a novel, holistic measure of the environmental risk people face globally, we test the spatial relationship between both types of pollution risks at the country level. We present the results of a prioritization assessment wherein the *Target* score reflects the relationships of *Vulnerability*, *Eco-health*, and *Readiness* for each country (see Methods for variable descriptions). This tool should allow policymakers and other stakeholders to place toxic pollution risk and climate impacts risk in dialogue with core structural aspects of a given country and not only identify where this risk is greatest, but also estimate where targeted actions in reducing this risk, and thus potential human suffering, might produce the highest return and probability of success when accounting for the institutional capacity of each country.

### Background

Human-produced pollution causes an array of cascading ecological and social effects [3,4,25,26]. These risks are pushing the Earth System into a planetary state distinct in human evolutionary history, posing substantial new risks and challenges [3,27–29]. Primary components of these changes and corresponding risks are toxic pollution—materials that are directly harmful to human health via emission into the air, water, or land—and non-toxic pollution—materials not directly toxic to humans but indirectly deleterious to human health via their emission as greenhouse gases (GHG) that change the climate and the functioning of the Earth System. Toxic pollution results in the death of more than 8 million people annually [7,9,30,31] and other harms to humans from cognitive dysfunction [32–39] to chronic respiratory illnesses [40–45]. Non-toxic pollution is driving global environmental changes that endanger human health through global warming [11,46–48], land degradation [28,49,50], extreme weather events [51,52], and sea-level rise [53,54]. The number of annual global deaths resulting from these changes is yet undetermined, but between 2030–2050 an additional 250,000 people are estimated to perish annually [55], and likely the actual figures are much higher [56]. By 2050 an estimated 530,000 additional deaths will occur annually solely due to food production losses caused by climate change [57]. The biogeophysical effects of human-produced pollution are not equally distributed, with low-income countries undergoing the highest risks and resultant negative human health impacts from toxic pollution and climate change [7–9,46]. Importantly, toxic pollution emissions primarily, though not solely, affect the areas more proximal to where they are released, whereas GHGs drive a global process of climatic change that can result in risk far from where they are emitted [10,11]. This difference is critical because lower-income countries are relatively more exposed to climate risk but middle- and high-income countries are responsible for a larger share of historical and contemporary GHG emissions [46,58–64].

The social outcomes and costs associated with these risk factors are as far reaching as the biogeophysical outcomes. The estimated current economic output lost due to the human health effects of toxic pollution is $4.6 trillion annually, or 6.2% of global GDP [9]. The estimated current economic productivity losses from global warming, in just the US and the EU, is $4 trillion. By 2100 world GDP per capita is expected to be reduced by 7.2% due to climate change [65]. By 2040 global climate change will add at least 20%—or $100 billion a year—to the annual global cost of extreme weather events [66]; in 2017 extreme weather exacerbated by climate change wrought $300 billion in just infrastructural and material damage alone [67]. The economic effects of climate change are not distributed equally across countries, with tropical countries at least 5% poorer than they would be in the absence of climate change [68] and increasing global income inequality within and between countries and classes, respectively, by approximately 25% [69]. Other indirect social effects from climate change range from the potential for increased rates of violent conflict and warfare [70–76] to increased incidence of mental health issues [77–80].

Low-income countries face relatively higher risks from toxic pollution and climate change than high-income countries [16,46]. However, the relationship *between* the global distribution of these two risks has not been explored and tested [9,23], resulting in a critical gap in current understandings of human health risk due to human-produced pollution. Further, where and how targeted efforts should be made to reduce or mitigate these risks with the highest rate and probability of return on efforts—i.e., reductions in the loss or risk of loss of human life—also remains unclear.

## Materials and methods

### Data

The processes and effects of human-produced toxic and non-toxic pollution are differentiable but interrelated. We collate and analyze three broadly utilized datasets (ND-GAIN, EPI, and GAHP) to measure the global distribution of risk from toxic pollution and climate change, to test the spatial relationship between these risks, and to identify how best to target their mitigation. All data utilized can be found in S1 Data and are publicly available from the institutions that curate them. All three datasets are for the year 2018, the most recent available data from all three sources that includes all of the same countries, at the time this article was written.

#### Notre Dame–Global Adaptation Index (ND-GAIN)

The ND-GAIN Country Index summarizes a country’s vulnerability and exposure to climate impacts risks and its readiness to improve climate resilience [12]. The full ND-GAIN index is comprised of 45 indicators of climate impacts risk (*Vulnerability*; 36 indicators) and *Readiness* (9 indicators) for 182 countries. ND-GAIN assesses the *Vulnerability* of a country by considering six life-supporting sectors: food, water, health, ecosystem services, human habitat and infrastructure. The exposure of each sector to climate-related or climate-exacerbated hazards, the sensitivity of that sector to the impacts of the hazard and the adaptive capacity of the sector to cope or adapt to these impacts, are in turn measured by six indicators each. ND-GAIN assesses *Readiness* by considering a country’s ability to implement adaptation actions if effectively incentivized. ND-GAIN measures overall *Readiness* by considering three components: economic readiness, governance readiness, and social readiness. ND-GAIN does not include measures for toxic pollution, nor does it measure GHG emissions rates; it measures only a country’s climate risk—their exposure and vulnerability to climate-driven hazards—and their readiness to deal with that risk.

#### Environmental Performance Index (EPI)

The EPI ranks 180 countries on 24 performance indicators across ten issue categories covering environmental health (*Eco-health*) and ecosystem vitality [16]. *Eco-health* measures environmental degradation and exposure to toxic pollution, while ecosystem vitality focuses on ecological health—such as tree cover loss—and the policies and protections in place to preserve it—e.g. national level biome protections. Ecosystem vitality includes measures that both partially overlap with ND-GAIN measures and measures of GHG emissions and other components not relevant to toxic pollution. As such we only utilize *Eco-health* from EPI, which represents toxic pollution distributions only. *Eco-health* measures toxic pollution risk via air quality (52%), water and sanitation (12%), and heavy metals (2%). The air quality measure focuses primarily on PM2.5 exposure and exceedances. While not the only hazardous air pollution, PM2.5 has been demonstrated to pose the greatest risk to human health due to effect and distribution [31,39,43,81–84]. The water and sanitation variable measures drinking water quality and sanitation infrastructure with regards to exposure to toxic pollutants, while the heavy metals measure is comprised solely of estimated lead exposure. Notably, many toxic pollutantsthat directly and pervasively harm humans are not included in *Eco-health*—e.g., air pollutants such as ground-level Ozone and Sulfur dioxide and heavy metals such as mercury and chromium. This is mostly due to lack of reliable and accessible data on the global distribution of these pollutants and because PM2.5 accounts for the majority of human health harm from air pollution [9,30,31].

#### Global Alliance on Health and Pollution (GAHP)

The GAHP estimates the number of toxic pollution deaths for a country [9]. They include deaths caused by exposure to toxic air, water, soil, and chemical pollution globally. The GAHP utilizes the Institute for Health Metric’s Global Burden of Disease Study [85] that measures annual deaths due to all causes. The GAHP results are conservative, as many known toxins are not included in their analysis, yet they assess toxic pollution to be the single largest cause of premature mortality. Their estimates indicate that in 2017 toxic pollution resulted in the premature death of at least 8.3 million premature deaths, or 15% of all deaths globally, and 275 million Disability-Adjusted Life Years. These deaths were caused by toxic air pollution (4.9 million; 59%), water pollution (1.6 million; 19%), occupational exposure (800,000; 9%), or lead exposure (1 million; 12%). As research on the effects of other pollutants becomes more certain—such as growing work on PFAS [86,87] and microplastics [88,89]—the number of premature deaths attributed to pollution is expected to increase substantially [7–9]. We operationalize their data, specifically the estimated proportion of total mortality due to pollution (*Proportion Mortality*) to test the relationship between the EPI assessed toxic pollution distribution (*Eco-health*) and deaths resulting from toxic pollution exposure, and thus validate ecosystem health as an effective measure of toxic pollution risk. We use *Proportion Mortality* as opposed to total pollution mortality or pollution mortality rate per 100,000 persons to avoid issues of country demographic effects such as total population, access to health care services, and other potentially confounding factors.

### Hypothesis

We hypothesize that there is a significant positive relationship between the spatial distribution of toxic environments (*Eco-health*) and climate risk (*Vulnerability*). Research strongly suggests that low- and middle-income countries face higher climate risks—though they contribute less than high-income countries to producing the risk—and are on average more impacted by toxic pollution. But, the alignment between the countries most and least at risk of each risk type, and the strength of this potential relationship, remains untested.

### Statistical analysis

To test the relationship between a country’s climate risk and toxic pollution exposure, first we employed Spearman’s Rank-Order Correlation Coefficient (r_s_) test to the relationship between the variables *Vulnerability*, *Eco-health*, and *Proportion Mortality*. To test the sensitivity of the results we tested the ratio values and the ranked values, as well as employed Pearson’s Correlation Coefficient (r) test. The results remained robust and relatively unchanged. We report the Spearman test results here because the rank order values provide a more easily interpreted and meaningful point of comparison between countries. We then repeated the correlation tests to include *Readiness* to explore the relationship between a countries readiness to adapt to or reduce climate risks and their respective pollution risks. Finally, we produced an estimate (*Target*) of where efforts in pollution risk mitigation have the highest probability of, and rate of, returns defined by the equation:

Target=((Vulnerability+Eco‐health)*Readiness)/100

We specify *Vulnerability* and *Eco-health* as additive and *Readiness* as multiplicative. Together *Vulnerability* and *Eco-health* account for where people face the highest pollution risks, while *Readiness* balances a country’s ability to do something about it if mitigation incentives or policies were targeted there. *Target*, though it results in a normally distributed measure (Anderson-Darling = 0.563; p = 0.143), is then best interpreted in rank order to compare countries. Research indicates that formal and informal institutional capacity is the most critical component for effective environmental management and regulation [90–99]. Thus, we weight *Readiness* (making it multiplicative as opposed to additive) to account for the effect of institutional capacity on pollution risk reduction outcomes. *Vulnerability* and eco-health are weighted equally as there is not a clear consensus as to whether it is toxic or non-toxic pollution that poses the greatest risk to human health. Note that to operationalize *Vulnerability* and *Eco-health* in this way, both variables were transformed in the dataset from their raw values to a 100-point scale and such that larger values represent worse health and greater vulnerability and lower values represent better health and lesser vulnerability (see S1 Data).

### Limitations

The data we utilize to account for toxic and non-toxic pollution do not measure all forms of harm or potential risks from these processes. For example, *Eco-health* only includes PM2.5 to assess air quality, whereas there are numerous hazardous air pollutants. As more and better data on other pollutants becomes available, it will need to be integrated into this assessment and the estimates of highest risk areas may potentially shift. Similar limitations are present in *Vulnerability* as a measure of climate impacts risk. The total toxic pollution death estimates that comprise *Proportion Mortality* include only those deaths where pollution-as-cause has high scientific consensus [7]. The authors estimate that this is only a relatively small portion of the premature mortality that toxic pollution causes or contributes to [7,9]. Also, our measures are static whereas the processes that they measure are dynamic. As such, our estimates provide a critical assessment of the global distribution of the combined risk of toxic pollution and climate impacts risk today, but they are temporally limited. Finally, the data are aggregated to the country level, when toxic and non-toxic pollution risks, as well as the readiness to address these risks, can vary widely within countries, and targeting decisions to reduce pollution may thus require finer intra-country assessments.

## Results

A strong (r_s_ = -0.798; 95% CI -0.852, -0.727) and statistically significant (p<0.0001) relationship exists between the spatial distribution of global climate risk (*Vulnerability*) and toxic pollution (*Eco-health*) (S1 Fig). Countries that are most at risk of the impacts of climate change are most often also the countries facing the highest risks of toxic pollution. Corroborating this finding, *Eco-health* and *Proportion Mortality* are strongly (r_s_ = -.793; 95% CI -0.848, -0.720) and significantly (p<0.0001) correlated; and *Proportion Mortality* and *Vulnerability* are strongly (r_s_ = 0.761; 95% CI 0.680, 0.823) and significantly (p<0.0001) correlated. In short, deaths resulting from toxic pollution are highest where the distribution of toxic pollution is greatest and, critically, also where the impacts of climate change pose the greatest risk.

**Fig 1:**
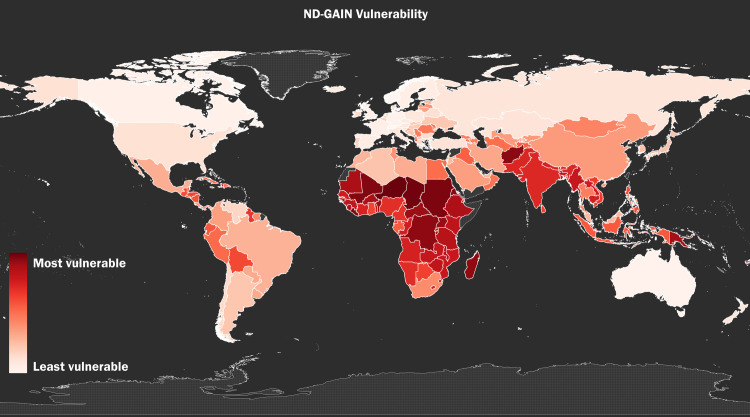
The global distribution of climate impacts risk. The global distribution of climate impacts risk using by-country rank-order, as measured by the variable *Vulnerability* from the 2018 ND-GAIN Country Index [12].

**Fig 2:**
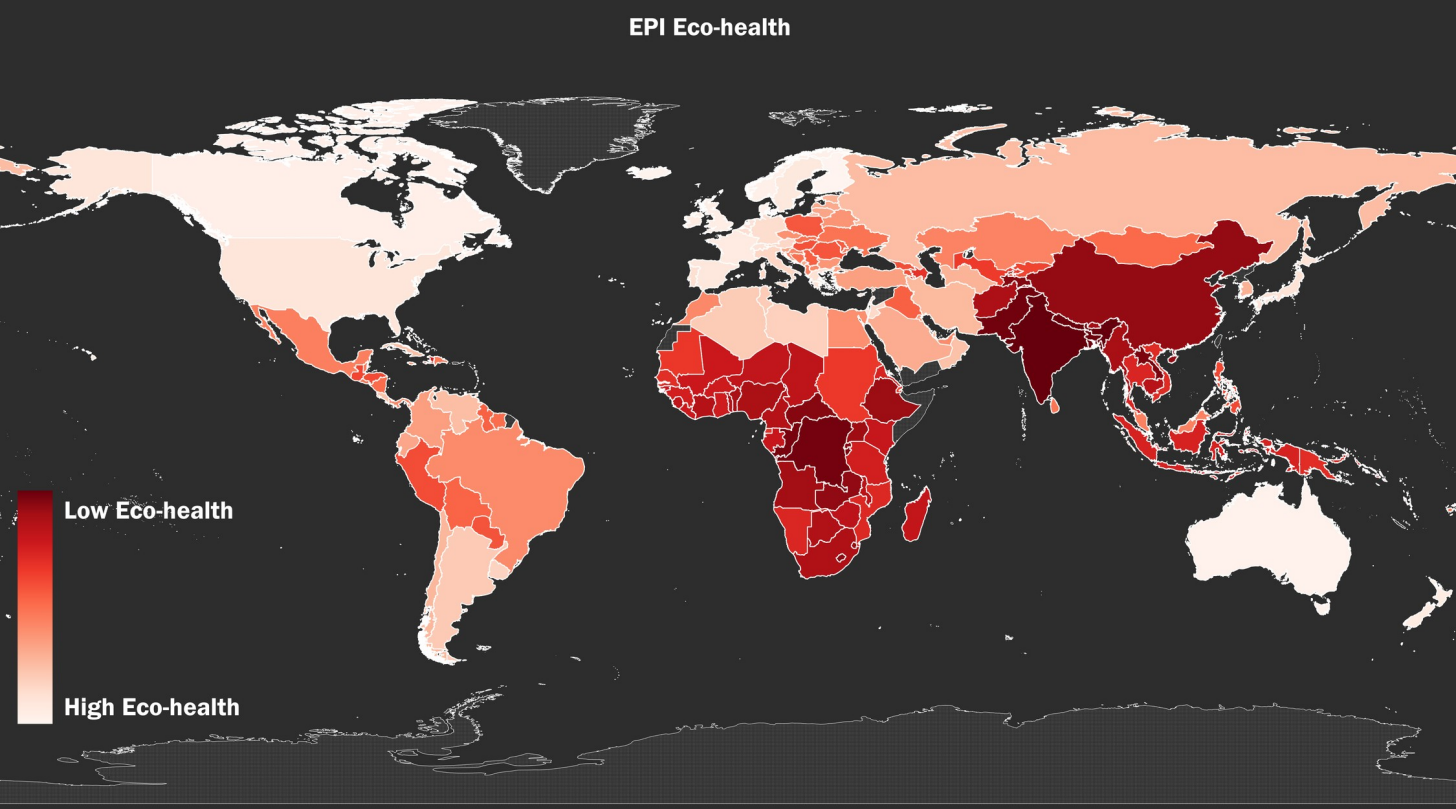
The global distribution of toxic pollution risk. The global distribution of toxic pollution risk using by-country rank-order, as measured by the variable *Eco-health* from the 2018 EPI [13].

**Fig 3:**
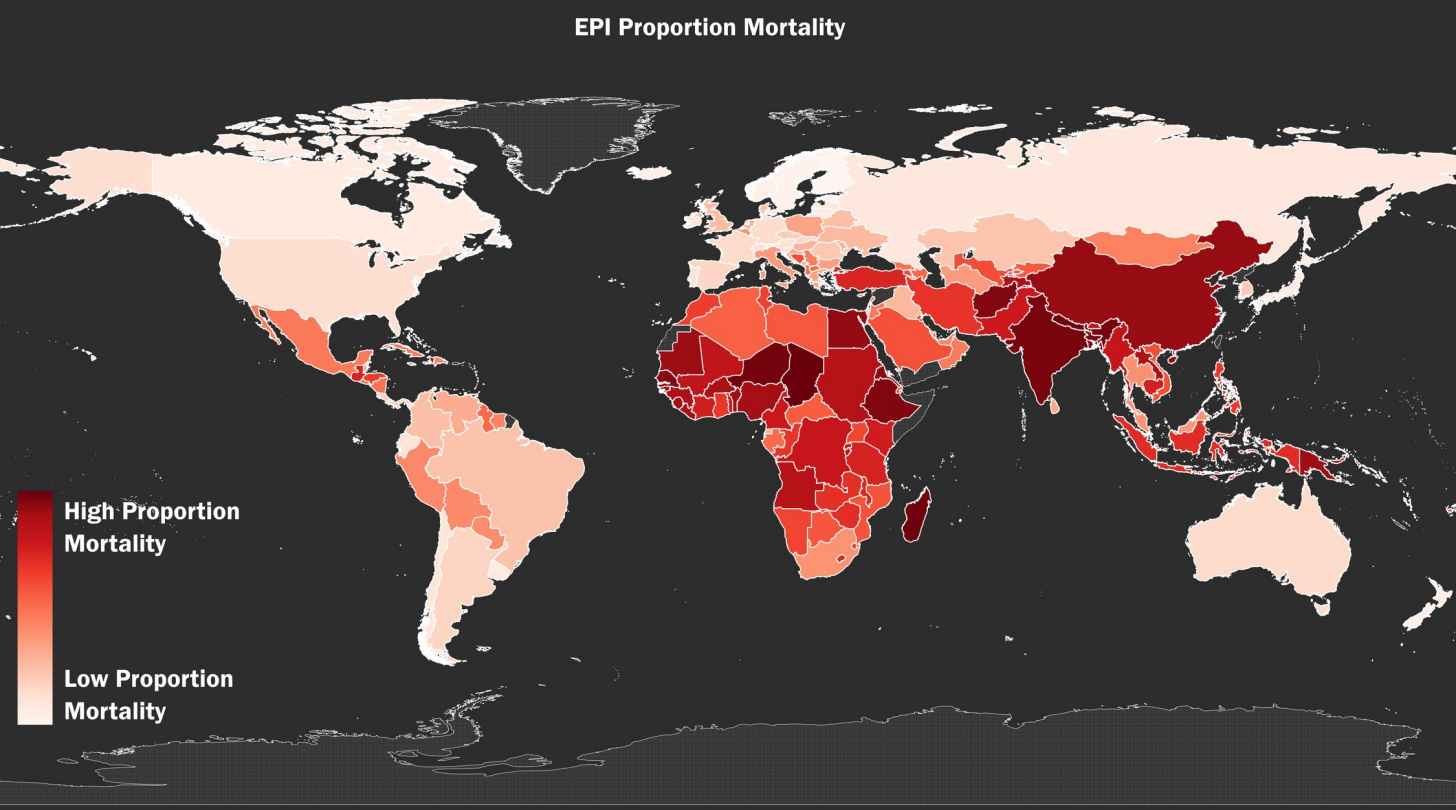
The global distribution of toxic pollution deaths. The global distribution of toxic pollution deaths as a percentage of total deaths in 2018, using by-country rank-order, as measured by the variable *Proportion Mortality* from the GAHP Pollution and Health Metrics Report [9].

**Fig 4:**
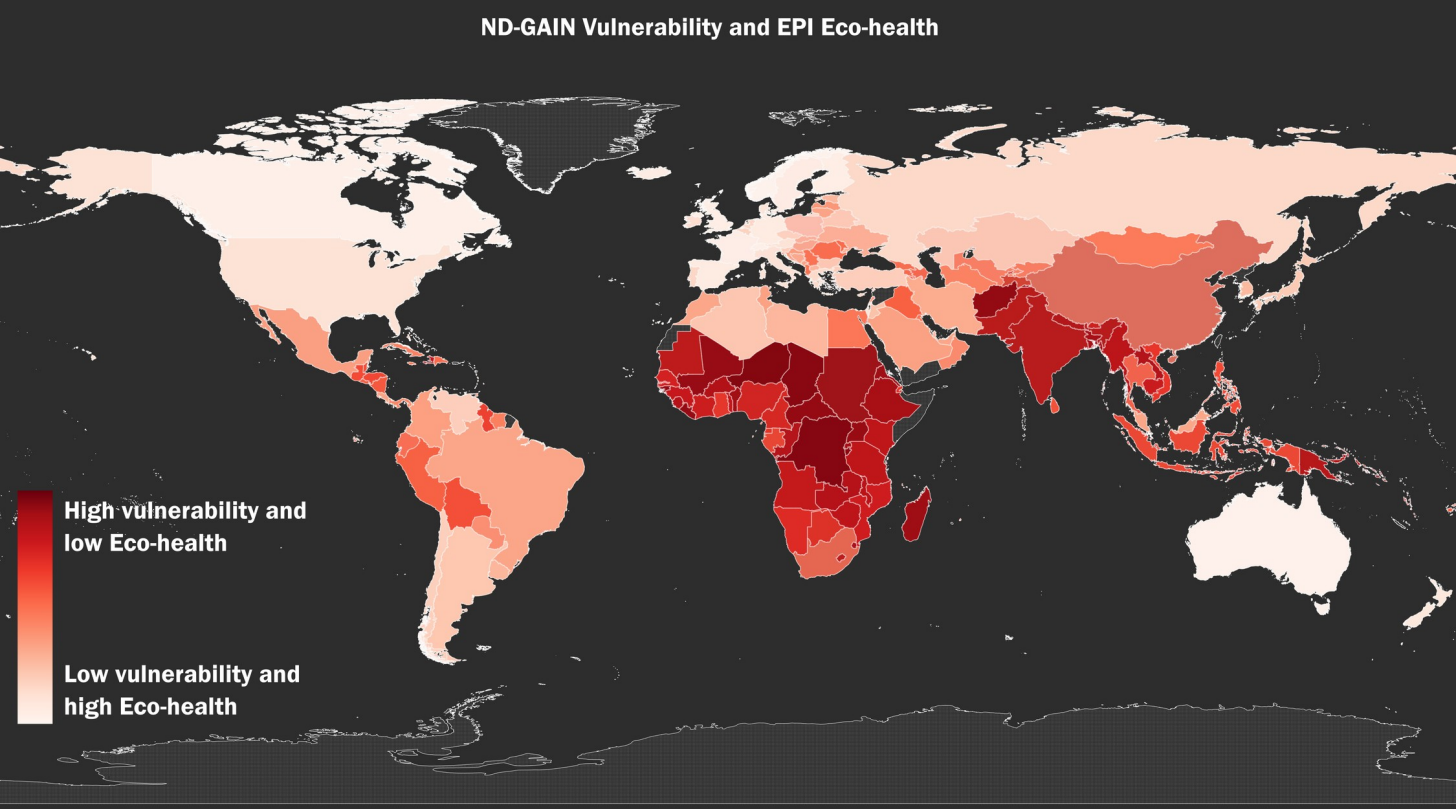
The global distribution of the combined toxic-climate risk. The global distribution of the combined risk of toxic pollution (low or high ecosystem health) and climate impacts (high or low vulnerability) risk using the average of the by-country rank-order of *Vulnerability* and *Eco-health*.

Figs 1–3 show the global distribution of *Vulnerability*, *Eco-health*, and *Proportion Mortality*, respectively. Fig 4 shows the combined global distribution of climate risk and toxic pollution risk (*Vulnerability* and *Eco-health* taken together); in alignment with our Spearman rank-order correlation coefficient results, Fig 4 depicts the equally weighted and averaged rank-order of *Vulnerability* and *Eco-health*, as opposed to the raw index values. As the correlation test results indicate and the maps visually affirm, the highest climate and toxic pollution risks appear to coincide in the same countries, and they are geographically concentrated across the African continent and Southeast Asia.

**Fig 5:**
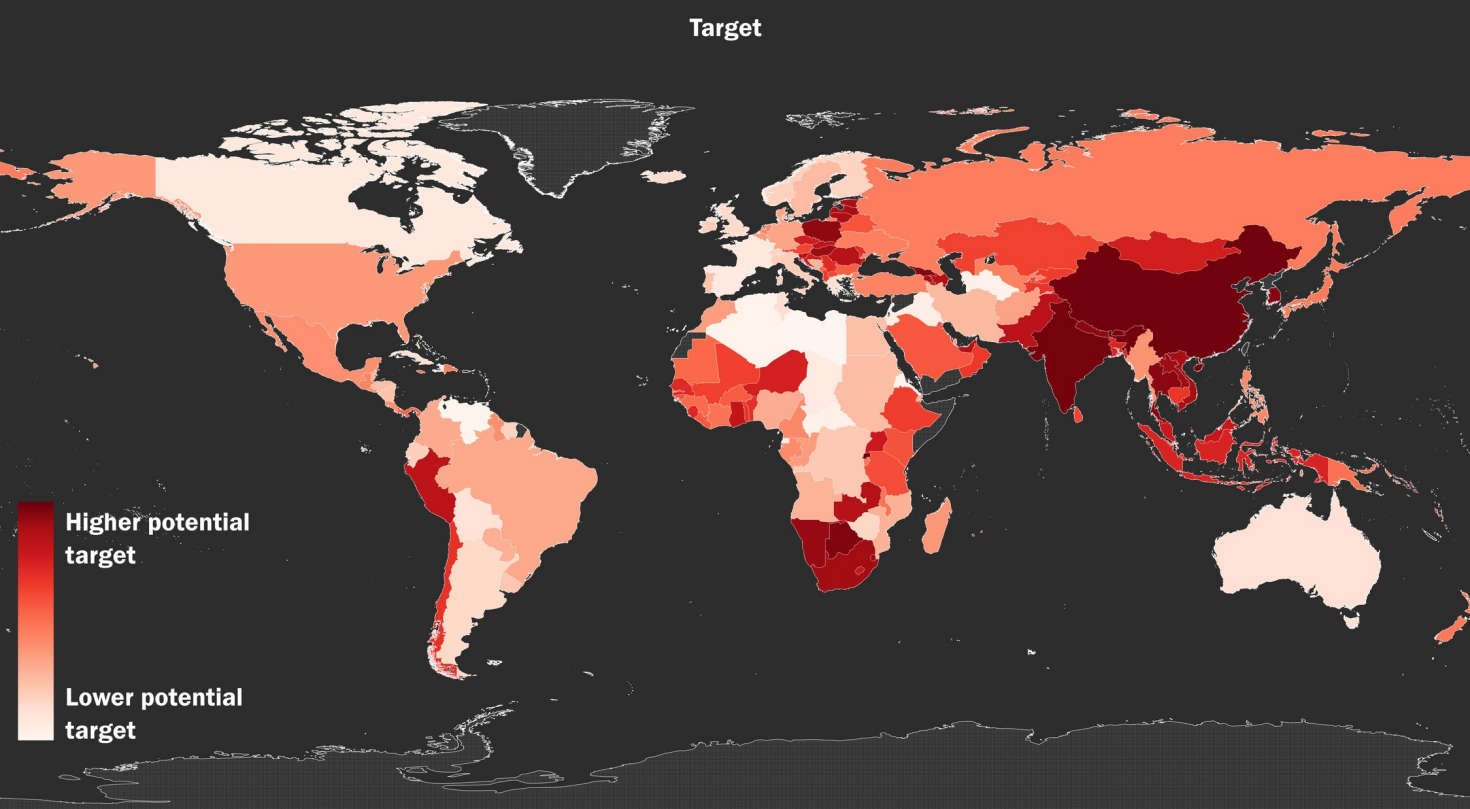
The global distribution of *Target*. The global distribution of *Target*, a measure of a country’s climate impacts risk, toxic pollution risk, and their potential readiness to mitigate these risks.

Our final analysis offers suggestions for how to reduce climate and toxic pollution risks. Directly targeting directly pollution reduction makes sense in countries that are assessed to have high *Readiness—*a measure of a country’s economic, governance, and social readiness to adapt to environmental risks—scores and will respond effectively to investments or other incentives, such as favorable trade terms. However, in places experiencing governance challenges, such as low state capacity or high corruption, efforts and resources might be better placed in addressing the governance challenges first. We therefore join *Vulnerability* and *Eco-health* with *Readiness* to produce *Target*. Table 1 lists the top 10 countries most likely to generate a high rate of return on effort in the reduction of toxic pollution and climate change impacts and the bottom 10 countries most likely to require attention to governance issues before pollution could be effectively addressed. Fig 5 depicts the global distribution of the *Target* variable results.

**Table 1:** The Top 10 and Bottom 10 countries to *Target* to reduce global toxic and non-toxic pollution risk.

Top 10 Countries	
Country Name	Target Rank
Singapore	1
Rwanda	2
China	3
India	4
Solomon Islands	5
Bhutan	6
Botswana	7
Georgia	8
Korea, Republic of	9
Thailand	10
Bottom 10 Countries	
Country Name	Target Rank
Equatorial Guinea	168
Iraq	169
Jordan	170
Lebanon	171
Central African Republic	172
Turkmenistan	173
Algeria	174
Eritrea	175
Venezuela	176
Libyan Arab Jamahiriya	177

The countries listed are ranked using the *Target* results which assesses the highest probability, and rate, of return on efforts to reduce toxic pollution and climate impacts risk.

## Discussion

Our tests of the relationship between the distribution of *Vulnerability* and *Eco-health* strongly indicate that the countries that face the greatest human health risks from toxic pollution are also those most at risk of the impacts wrought by our changing climate. The potential for these two types of risk to interact and synergize in the environment and in the human body, while understudied [9,15,100], is substantial [21,101,102]. Compounding these risks is the fact that the countries most at-risk include many of the poorest and least developed in the world, such as the Democratic Republic of the Congo (DRC) (#1), Burundi (#2), and the Central African Republic (#3), which are ranked 179^th^, 185^th^, and 188^th^ of 189, respectively, by the United Nations Human Development Index [103]. The demographic, ecological, and social factors that contribute to these consistently ‘low’ rankings are not independent of each other and are demonstrative of broader patterns of inequality [68,69,104–107]. Our results affirm the indications from previous research that low-income countries face the highest risks of impacts from climate change and risk from toxic pollution. Our results do not estimate the corresponding impact of the interactions of toxic and climate risk, but evidence from other studies suggests the combined effect is substantial [101,108,109]. For example, warming temperatures have been shown to both increase rates of heat-related illness and mortality and to produce and to enhance the toxicity of contaminants in the environment [20,48,110]Thus, targeting the improvement of conditions, outcomes, and structures in such countries is globally beneficial and practically and ethically urgent. However, not all countries offer the same basal context for such actions and thus models for assessing structures of risk reduction efforts remain critical.

The incidence of toxic pollution and climate impacts risk in low-income countries is shaped by intersecting local or domestic structural conditions—including reduced capacity for environmental policy regulation and enforcement, especially as applied to industry and transportation standards [111], as well as physical geography—and by ‘external’ factors such as foreign firms taking advantage of reduced environmental regulation [112] or shifts in precipitation patterns driven by climate change [46]. For example, the DRC faces the intersecting risks of PM2.5 transported from the Saharan Desert by predominant wind currents from the northeast (external) but also locally produced PM2.5 from low grade fuels and inefficient vehicles concentrated in city centers, and a landscape littered with foreign commercial (external) and local artisanal (internal) extractive mining operations that alter landscapes and pollute waterways with heavy metals [113–115]. Intersecting with these factors are climate impact risks such as changing rainfall patterns and increased warming that disrupt the farming cycle and concurrently increase risks of malnutrition and incidence of communicable and non-communicable disease [116]. A similar pattern of intersecting, mutually amplifying environmental risks and endogenous and exogenous factors can be found in all of the countries at the top of our combined *Vulnerability*-*Eco-health* list. Notably, our results find that the top one-third of countries at risk of toxic pollution and climate impacts represent more than two-thirds of the world’s population, highlighting the magnitude of the problem and unequal distribution of environmental risk.

Given that a large portion of the world’s population lives in countries at higher toxic pollution and climate impacts risk, understanding where and how to target in pollution risk mitigation is critical to maximizing reductions of potential human harm. Our *Target* results indicate that the countries facing the highest toxic pollution and climate impacts risk also often lack institutional *Readiness* to address these problems. This does not mean that efforts to support these countries or to improve their conditions should be abandoned—or that efforts to restrict the external factors acting upon them should be altered—but rather that focusing on and bolstering institutional capacity development is likely the initial, and primary, focus of effort.

However, many other countries could benefit from direct efforts towards pollution risk reduction. Two of the countries in our top 5 highest *Target* results, China and India, are substantially economically developed and hold prominent geopolitical power. They are the largest countries on the planet, together representing over 2.5 billion people, and both have relatively high *Proportion Mortality* ranks, ranking 13^th^ and 5^th^ (17.9% and 23.5% of annual deaths are associated with toxic pollution, equaling 1.9 and 2.3 million premature deaths annually, respectively) [9]. Despite these high rates, recently developed national policies and their resultant impacts suggest that the benefits of augmenting facilitation of such changes (via targeted policy efforts and incentives) are large and achievable. For example, as a result of China’s Air Pollution and Prevention and Control Action Plan enacted in 2013 and targeting PM2.5 specifically, Han et al. [117] find that annual average PM2.5 levels reduced substantially between 2013–2017—from 101.7 μg m^-3^ to 58.6 μg m^-3^, a 40% reduction. The improved air quality resulted in significant corresponding reductions in respiratory disease and cardiovascular disease mortality rates—both of which have been demonstrated to be strongly linked to toxic air pollution [30,31,84]. Interestingly, if global annual average PM2.5 emissions could be reduced even half the amount that China achieved—i.e. 20%—the estimated corresponding effect would be 1.4 million fewer premature deaths annually worldwide [31]. Han et al. caution that air pollution remains severe across China and further regulatory restrictions and mitigation efforts are much needed. Importantly, while our results indicate that targeting these countries will likely produce positive reductions in human suffering domestically, as China is the world’s leading total GHG emitter and India is on track to join it at the top in the future, both emit high rates of toxic pollution (*Eco-health)* that also can travel outside of their borders. Thus, there is strong potential for co-benefits from efforts focused on China and India in risk reduction for neighboring countries and other countries at-risk of climate change more broadly.

Importantly, our *Target* results should be understood as assessing where policy tools can potentially be leveraged with high effects—whether those tools be local, national, and international in nature, scale, and application. A range of measures can be used to promote risk reduction such as trade incentives, be they negative or positive incentives, or other policy and regulatory enforcement mechanisms. Likely a suite of tools will need to be employed and leveraged to achieve the desired risk reduction goals [8,11,17,94].

## Conclusion

Despite the interdependence of risks from toxic and non-toxic pollution, no prior study has analyzed the joint global distribution of these risks nor offered evidence-based arguments for how to address the co-impact of both risks efficiently. We fill components of these knowledge gaps with data on global climate risk, environmental quality, toxic pollution caused mortality, and institutional capacity for 176 countries. Our analysis demonstrates a strong correlation between toxic pollution risk and climate risk, along with varying capacities of countries to manage that risk. We argue that the *Target* assessment tool can be usefully employed to determine optimal locations for risk reduction, while also drawing attention to high-risk low-income countries that must urgently address governance challenges in order to have a chance at successfully addressing pollution risk.

Some have argued that the challenges presented by human-produced pollution may be nearing tipping points past which returning to the ecological niche representative of most of human history will become unattainable [3,28]. However, recent global events have highlighted that behavioral changes at national levels can have dramatic and rapid effects on pollution levels [118–120]. For example, regulation on movement and activity in response to Covid-19 resulted in dramatic decreases in air pollution rates in some countries [120,121]. Broad and expansive action, akin to that sparked by Covid-19 but more strategic and sustainable is needed to combat the pressures wrought by human-produced pollution on the human niche [46,50,122]. It is our hope that the *Target* measure can be deployed as part of a larger ongoing empirical assessment of human caused pollution to increase political and social will at national levels for drastic policy measures and broad reaching pollution reduction actions.

## Supporting information

S1 Fig Results of Spearman’s Rank-Order Correlation Coefficient (r_s_) and corresponding scatterplots for the variables *Vulnerability*, *Eco-health*, and *Proportion Mortality*. (TIF)

S1 Data (CSV)

## References

[1] Rockström J, Steffen W, Noone K, Persson Å, Chapin FS, Lambin E, et al. Planetary Boundaries: Exploring the Safe Operating Space for Humanity. Ecology and Society. 2009;14. Available: https://www.jstor.org/stable/26268316.

[2] Steffen W, Richardson K, Rockström J, Cornell SE, Fetzer I, Bennett EM, et al. Planetary boundaries: Guiding human development on a changing planet. Science. 2015;347: 1259855. doi: 10.1126/science.1259855 25592418

[3] Steffen W, Rockström J, Richardson K, Lenton TM, Folke C, Liverman D, et al. Trajectories of the Earth System in the Anthropocene. PNAS. 2018; 201810141. doi: 10.1073/pnas.1810141115 30082409 PMC6099852

[4] Dalby S. Anthropocene Formations: Environmental Security, Geopolitics and Disaster. Theory, Culture & Society. 2017;34: 233–252. doi: 10.1177/0263276415598629

[5] Biopolitics Dalby S. and climate security in the Anthropocene. Geoforum. 2013;49: 184–192. doi: 10.1016/j.geoforum.2013.06.013

[6] Dalby S. Firepower: Geopolitical Cultures in the Anthropocene. Geopolitics. 2018;23: 718–742. doi: 10.1080/14650045.2017.1344835

[7] Landrigan PJ, Fuller R, Acosta NJR, Adeyi O, Arnold R, Basu N (Nil), et al. The Lancet Commission on pollution and health. The Lancet. 2017;391: 462–512. doi: 10.1016/S0140-6736(17)32345-0 29056410

[8] Landrigan PJ, Fuller R, Hu H, Caravanos J, Cropper M, Hanrahan D, et al. Pollution and Global Health–An Agenda for Prevention. Environmental Health Perspectives. 2018;126: 1–6. doi: 10.1289/EHP3141 30118434 PMC6108842

[9] Fuller R, Sandilya K, Hanrahan D. Pollution and Health Metrics. New York, NY: Global Alliance on Health and Pollution; 2019 p. 56. Available: https://gahp.net/wp-content/uploads/2019/12/PollutionandHealthMetrics-final-12_18_2019.pdf.

[10] Watts N, Amann M, Ayeb-Karlsson S, Belesova K, Bouley T, Boykoff M, et al. The Lancet Countdown on health and climate change: from 25 years of inaction to a global transformation for public health. The Lancet. 2017;S0140-6736: 32464–9. doi: 10.1016/S0140-6736(17)32464-9 29096948

[11] Watts N, Amann M, Arnell N, Ayeb-Karlsson S, Belesova K, Boykoff M, et al. The 2019 report of The Lancet Countdown on health and climate change: ensuring that the health of a child born today is not defined by a changing climate. The Lancet. 2019;394: 1836–1878. doi: 10.1016/S0140-6736(19)32596-6 PMC7616843 31733928

[12] ND-GAIN. ND-GAIN Country Index rankings | ND-GAIN Index. Notre Dame: Notre Dame Global Adaptation Index; 2019. Available: http://index.gain.org/ranking.

[13] EPI. Environmental Performance Index 2018. New Haven, CT: Yale Center for Environmental Law and Policy; 2018 p. 200.

[14] Wild CP. The exposome: from concept to utility. Int J Epidemiol. 2012;41: 24–32. doi: 10.1093/ije/dyr236 22296988

[15] Vermeulen R, Schymanski EL, Barabási A-L, Miller GW. The exposome and health: Where chemistry meets biology. Science. 2020;367: 392–396. doi: 10.1126/science.aay3164 31974245 PMC7227413

[16] GAHP. Pollution Health Overview and Solutions. Geneva: Global Alliance on Health and Pollution; 2019 p. 5.

[17] Watts N, Adger N, Zhang Q, Gong P, Montgomery H, Costello A. Health and climate change: policy responses to protect public health. The Lancet. 2015; 386–914. doi: 10.1016/S0140-6736(15)60854-6 26111439

[18] Mulenga D, Siziya S. Indoor Air Pollution Related Respiratory Ill Health, a Sequel of Biomass Use. SciMedicine Journal. 2019;1: 30–37. doi: 10.28991/SciMedJ-2019-0101-5

[19] Schiedek D, Sundelin B, Readman JW, Macdonald RW. Interactions between climate change and contaminants. Marine Pollution Bulletin. 2007;54: 1845–1856. doi: 10.1016/j.marpolbul.2007.09.020 17963794

[20] Gmitrowicz-Iwan J, Ligęza S, Pranagal J, Kołodziej B, Smal H. Can climate change transform non-toxic sediments into toxic soils? Science of The Total Environment. 2020;747: 141201. doi: 10.1016/j.scitotenv.2020.141201 32777500

[21] Marcantonio RA, Field S, Regan PM. Toxic trajectories under future climate conditions. PLOS ONE. 2019;14: e0226958. doi: 10.1371/journal.pone.0226958 31869830 PMC6927791

[22] Marcantonio R, Field S, Regan PM. Toxicity travels in a changing climate. Environmental Science & Policy. 2020;114: 560–569. doi: 10.1016/j.envsci.2020.09.029

[23] Landrigan PJ, Fuller R, Haines A, Watts N, McCarthy G. Pollution prevention and climate change mitigation: measuring the health benefits of comprehensive interventions. The Lancet Planetary Health. 2018;2: e515–e516. doi: 10.1016/S2542-5196(18)30226-2 30526935

[24] Psaki J. Press Briefing by Press Secretary Jen Psaki, Special Presidential Envoy for Climate John Kerry, and National Climate Advisor Gina McCarthy, January 27, 2021. Washington, D.C.: The White House Press Briefings; 2021 Jan p. 14. Available: https://www.whitehouse.gov/briefing-room/press-briefings/2021/01/27/press-briefing-by-press-secretary-jen-psaki-special-presidential-envoy-for-climate-john-kerry-and-national-climate-advisor-gina-mccarthy-january-27-2021/.

[25] Steffen W, Crutzen PJ, McNeill JR. The Anthropocene: Are Humans Now Overwhelming the Great Forces of Nature. AMBIO: A Journal of the Human Environment. 2007;36: 614–621. doi: 10.1579/0044-7447(2007)36[614:taahno]2.0.co;2 18240674

[26] Ellis EC, Richerson PJ, Mesoudi A, Svenning J-C, Odling-Smee J, Burnside WR. Evolving the human niche. PNAS. 2016;113: E4436–E4436. doi: 10.1073/pnas.1609425113 27432994 PMC4978239

[27] Steffen W, Broadgate W, Deutsch L, Gaffney O, Ludwig C. The trajectory of the Anthropocene: the great acceleration. The Anthropocene Review. 2015;2: 81–98.

[28] Xu C, Kohler TA, Lenton TM, Svenning J-C, Scheffer M. Future of the human climate niche. PNAS. 2020;117: 11350–11355. doi: 10.1073/pnas.1910114117 32366654 PMC7260949

[29] Lenton TM, Rockström J, Gaffney O, Rahmstorf S, Richardson K, Steffen W, et al. Climate tipping points—too risky to bet against. Nature. 2019;575: 592–595. doi: 10.1038/d41586-019-03595-0 31776487

[30] Pope A, Lefler Jacob S., Ezzati Majid, Higbee Joshua D., Marshall Julian D., Kim Sun-Young, et al. Mortality Risk and Fine Particulate Air Pollution in a Large, Representative Cohort of U.S. Adults. Environmental Health Perspectives. 2020;127: 077007. doi: 10.1289/EHP4438 31339350 PMC6792459

[31] Burnett R, Chen H, Szyszkowicz M, Fann N, Hubbell B, Pope CA, et al. Global estimates of mortality associated with long-term exposure to outdoor fine particulate matter. PNAS. 2018;115: 9592–9597. doi: 10.1073/pnas.1803222115 30181279 PMC6156628

[32] Carey IM, Anderson HR, Atkinson RW, Beevers SD, Cook DG, Strachan DP, et al. Are noise and air pollution related to the incidence of dementia? A cohort study in London, England. BMJ Open. 2018;8: e022404. doi: 10.1136/bmjopen-2018-022404 30206085 PMC6144407

[33] Chen H, Kwong JC, Copes R, Tu K, Villeneuve PJ, Donkelaar A van, et al. Living near major roads and the incidence of dementia, Parkinson’s disease, and multiple sclerosis: a population-based cohort study. The Lancet. 2017;389: 718–726. doi: 10.1016/S0140-6736(16)32399-6 28063597

[34] Chen JC, Wang X, Wellenius GA, Serre ML, Driscoll I, Casanova R, et al. Ambient air pollution and neurotoxicity on brain structure: Evidence from women’s health initiative memory study. Ann Neurol. 2015;78: 466–476. doi: 10.1002/ana.24460 26075655 PMC4546504

[35] Wu Y-C, Lin Y-C, Yu H-L, Chen J-H, Chen T-F, Sun Y, et al. Association between air pollutants and dementia risk in the elderly. Alzheimers Dement (Amst). 2015;1: 220–228. doi: 10.1016/j.dadm.2014.11.015 27239507 PMC4876896

[36] Calderón-Garcidueñas L, Gónzalez-Maciel A, Reynoso-Robles R, Delgado-Chávez R, Mukherjee PS, Kulesza RJ, et al. Hallmarks of Alzheimer disease are evolving relentlessly in Metropolitan Mexico City infants, children and young adults. APOE4 carriers have higher suicide risk and higher odds of reaching NFT stage V at ≤40 years of age. Environmental Research. 2018;164: 475–487. doi: 10.1016/j.envres.2018.03.023 29587223

[37] Calderón-Garcidueñas L, Engle R, Mora-Tiscareño A, Styner M, Gómez-Garza G, Zhu H, et al. Exposure to severe urban air pollution influences cognitive outcomes, brain volume and systemic inflammation in clinically healthy children. Brain Cogn. 2011;77: 345–355. doi: 10.1016/j.bandc.2011.09.006 22032805

[38] Maher BA, Ahmed IAM, Karloukovski V, MacLaren DA, Foulds PG, Allsop D, et al. Magnetite pollution nanoparticles in the human brain. PNAS. 2016;113: 10797–10801. doi: 10.1073/pnas.1605941113 27601646 PMC5047173

[39] Shehab MA, Pope FD. Effects of short-term exposure to particulate matter air pollution on cognitive performance. Sci Rep. 2019;9: 1–10. doi: 10.1038/s41598-018-37186-2 31160655 PMC6546704

[40] Clark NA, Demers PA, Karr CJ, Koehoorn M, Lencar C, Tamburic L, et al. Effect of Early Life Exposure to Air Pollution on Development of Childhood Asthma. Environmental Health Perspectives. 2010;118: 284–290. doi: 10.1289/ehp.0900916 20123607 PMC2831931

[41] Gehring U, Wijga AH, Hoek G, Bellander T, Berdel D, Brüske I, et al. Exposure to air pollution and development of asthma and rhinoconjunctivitis throughout childhood and adolescence: a population-based birth cohort study. The Lancet Respiratory Medicine. 2015;3: 933–942. doi: 10.1016/S2213-2600(15)00426-9 27057569

[42] Guarnieri M, Balmes JR. Outdoor air pollution and asthma. The Lancet. 2014;383: 1581–1592. doi: 10.1016/S0140-6736(14)60617-6 24792855 PMC4465283

[43] Anenberg SC, Horowitz LW, Tong DQ, West JJ. An Estimate of the Global Burden of Anthropogenic Ozone and Fine Particulate Matter on Premature Human Mortality Using Atmospheric Modeling. Environ Health Perspect. 2010;118: 1189–1195. doi: 10.1289/ehp.0901220 20382579 PMC2944076

[44] Mikati I, Benson AF, Luben TJ, Sacks JD, Richmond-Bryant J. Disparities in Distribution of Particulate Matter Emission Sources by Race and Poverty Status. Am J Public Health. 2018;108: 480–485. doi: 10.2105/AJPH.2017.304297 29470121 PMC5844406

[45] Sarkar C, Zhang B, Ni M, Kumari S, Bauermeister S, Gallacher J, et al. Environmental correlates of chronic obstructive pulmonary disease in 96 779 participants from the UK Biobank: a cross-sectional, observational study. The Lancet Planetary Health. 2019;3: e478–e490. doi: 10.1016/S2542-5196(19)30214-1 31777339

[46] IPCC. Summary for Policy Makers. In Global warming of 1.5°C. An IPCC special report on the impacts of global warming of 1.5°C above pre-industrial levels and related global greenhouse gas emission pathways. Geneva, Switzerland: World Meteorological Organization; 2018.

[47] Bastin J-F, Clark E, Elliott T, Hart S, Hoogen J van den, Hordijk I, et al. Understanding climate change from a global analysis of city analogues. PLOS ONE. 2019;14: e0217592. doi: 10.1371/journal.pone.0217592 31291249 PMC6619606

[48] Mora C, Dousset B, Caldwell IR, Powell FE, Geronimo RC, Bielecki CR, et al. Global risk of deadly heat. Nature Climate Change. 2017;7: 501–506. doi: 10.1038/nclimate3322

[49] Ellis EC. Why Is Human Niche Construction Transforming Planet Earth? Molding the Planet. 2016; 63–70.

[50] IPCC. Climate Change and Land: An IPCC Special Report on climate change, desertification, land degradation, sustainable land management, food security, and greenhouse gas fluxes in terrestrial ecosystems. Geneva, Switzerland: International Panel on Climate Change; 2019 p. 43.

[51] Mann ME, Rahmstorf S, Kornhuber K, Steinman BA, Miller SK, Coumou D. Influence of Anthropogenic Climate Change on Planetary Wave Resonance and Extreme Weather Events. Scientific Reports. 2017;7: 45242. doi: 10.1038/srep45242 28345645 PMC5366916

[52] Cornwall E. Efforts to link climate change to severe weather gain ground. Science. 2016;351: 1249–1252. doi: 10.1126/science.351.6279.1249 26989227

[53] IPCC. IPCC Special Report on the Ocean and Cryosphere in a Changing Climate. New York: International Panel on Climate Change; 2019 p. 1170.

[54] Taherkhani M, Vitousek S, Barnard PL, Frazer N, Anderson TR, Fletcher CH. Sea-level rise exponentially increases coastal flood frequency. Sci Rep. 2020;10: 1–17. doi: 10.1038/s41598-019-56847-4 32300112 PMC7162943

[55] WHO. Quantitative risk assessment of the effects of climate change on selected causes of death, 2030s and 2050s. Geneva: World Health Organization; 2014. Available: https://apps.who.int/iris/handle/10665/134014.

[56] Haines A, Ebi K. The Imperative for Climate Action to Protect Health. New England Journal of Medicine. 2019;380: 263–273. doi: 10.1056/NEJMra1807873 30650330

[57] Springmann M, Mason-D’Croz D, Robinson S, Garnett T, Godfray HCJ, Gollin D, et al. Global and regional health effects of future food production under climate change: a modelling study. The Lancet. 2016;387: 1937–1946. doi: 10.1016/S0140-6736(15)01156-3 26947322

[58] Oswald Y, Owen A, Steinberger JK. Large inequality in international and intranational energy footprints between income groups and across consumption categories. Nat Energy. 2020;5: 231–239. doi: 10.1038/s41560-020-0579-8

[59] Matthews HD. Quantifying historical carbon and climate debts among nations. Nature Climate Change. 2016;6: 60–64. doi: 10.1038/nclimate2774

[60] Matthews HD, Graham TL, Keverian S, Lamontagne C, Seto D, Smith TJ. National contributions to observed global warming. Environ Res Lett. 2014;9: 014010. doi: 10.1088/1748-9326/9/1/014010

[61] Otto FEL, Skeie RB, Fuglestvedt JS, Berntsen T, Allen MR. Assigning historic responsibility for extreme weather events. Nature Climate Change. 2017;7: 757–759. doi: 10.1038/nclimate3419

[62] den Elzen MGJ, Olivier JGJ, Höhne N, Janssens-Maenhout G. Countries’ contributions to climate change: effect of accounting for all greenhouse gases, recent trends, basic needs and technological progress. Climatic Change. 2013;121: 397–412. doi: 10.1007/s10584-013-0865-6

[63] den Elzen M, Fuglestvedt J, Höhne N, Trudinger C, Lowe J, Matthews B, et al. Analysing countries’ contribution to climate change: scientific and policy-related choices. Environmental Science & Policy. 2005;8: 614–636. doi: 10.1016/j.envsci.2005.06.007

[64] Ward DS, Mahowald NM. Contributions of developed and developing countries to global climate forcing and surface temperature change. Environ Res Lett. 2014;9: 074008. doi: 10.1088/1748-9326/9/7/074008

[65] Kahn ME, Mohaddes K, Ng RNC, Pesaran MH, Raissi M, Yang J-C. Long-Term Macroeconomic Effects of Climate Change: A Cross-Country Analysis. National Bureau of Economic Research; 2019 Aug. Report No.: 26167. doi: 10.3386/w26167

[66] Cambridge Centre for Risk Studies. Global Risk Index 2019. University of Cambridge: Cambridge Centre for Risk Studies; 2019 p. 16. Available: https://www.jbs.cam.ac.uk/fileadmin/user_upload/research/centres/risk/downloads/crs-global-risk-index-exec-summary-2019.pdf.

[67] Smith A, Lott N, Houston T, Shein K, Crouch J, Enloe J. U.S. Billion-Dollar Weather & Climate Disasters 1980–2018. Silver Springs, Maryland: National Oceanic and Atmospheric Administration; 2018 pp. 1–14.

[68] Burke M, Tanutama V. Climatic Constraints on Aggregate Economic Output. Cambridge, MA: National Bureau of Economic Research; 2019 Apr p. w25779. Report No.: w25779. doi: 10.3386/w25779

[69] Diffenbaugh NS, Burke M. Global warming has increased global economic inequality. Proc Natl Acad Sci USA. 2019;116: 9808–9813. doi: 10.1073/pnas.1816020116 31010922 PMC6525504

[70] Hsiang S, Burke M. Climate, conflict, and social stability: what does the evidence say? Climatic Change. 2014;123: 39–55. doi: 10.1007/s10584-013-0868-3

[71] Hsiang S, Burke M, Miguel E. Quantifying the Influence of Climate on Human Conflict. Science. 2013;341: 1235367–1235367. doi: 10.1126/science.1235367 24031020

[72] Hendrix CS, Haggard S. Global food prices, regime type, and urban unrest in the developing world. Journal of Peace Research. 2015;52: 143–157. doi: 10.1177/0022343314561599

[73] Hendrix CS, Salehyan I. Climate change, rainfall, and social conflict in Africa. Journal of Peace Research. 2012;49: 35–50. doi: 10.1177/0022343311426165

[74] Mach KJ, Kraan CM, Adger N, Buhaug H, Burke M, Fearon JD, et al. Climate as a risk factor for armed conflict. Nature. 2019;571: 193–197. doi: 10.1038/s41586-019-1300-6 31189956

[75] Kelley CP, Mohtadi S, Cane MA, Seager R, Kushnir Y. Climate change in the Fertile Crescent and implications of the recent Syrian drought. Proceedings of the National Academy of Sciences. 2015;112: 3241–3246. doi: 10.1073/pnas.1421533112 25733898 PMC4371967

[76] Fagan B. The Great Warming: Climate Change and the Rise and Fall of Civilizations. Bloomsbury Publishing USA; 2010.

[77] Berry HL, Bowen K, Kjellstrom T. Climate change and mental health: a causal pathways framework. International Journal of Public Health. 2010;55: 123–132. doi: 10.1007/s00038-009-0112-0 20033251

[78] Cunsolo Willox A, Stephenson E, Allen J, Bourque F, Drossos A, Elgarøy S, et al. Examining relationships between climate change and mental health in the Circumpolar North. Regional Environmental Change. 2015;15: 169–182. doi: 10.1007/s10113-014-0630-z

[79] Willox AC, Harper SL, Edge VL, Landman K, Houle K, Ford JD. The land enriches the soul: On climatic and environmental change, affect, and emotional health and well-being in Rigolet, Nunatsiavut, Canada. Emotion, Space and Society. 2013;6: 14–24. doi: 10.1016/j.emospa.2011.08.005

[80] Bourque F, Willox A. Climate change: The next challenge for public mental health? International Review of Psychiatry. 2014;26: 415–422. doi: 10.3109/09540261.2014.925851 25137107

[81] Silva RA, West JJ, Zhang Y, Anenberg SC, Lamarque J-F, Shindell DT, et al. Global premature mortality due to anthropogenic outdoor air pollution and the contribution of past climate change. Environ Res Lett. 2013;8: 034005. doi: 10.1088/1748-9326/8/3/034005

[82] Cohen AJ, Brauer M, Burnett R, Anderson HR, Frostad J, Estep K, et al. Estimates and 25-year trends of the global burden of disease attributable to ambient air pollution: an analysis of data from the Global Burden of Diseases Study 2015. The Lancet. 2017;389: 1907–1918. doi: 10.1016/S0140-6736(17)30505-6 28408086 PMC5439030

[83] Vodonos A, Awad YA, Schwartz J. The concentration-response between long-term PM2.5 exposure and mortality; A meta-regression approach. Environmental Research. 2018;166: 677–689. doi: 10.1016/j.envres.2018.06.021 30077140

[84] Wu X, Braun D, Schwartz J, Kioumourtzoglou MA, Dominici F. Evaluating the impact of long-term exposure to fine particulate matter on mortality among the elderly. Science Advances. 2020; 13. doi: 10.1126/sciadv.aba5692 32832626 PMC7439614

[85] James SL, Abate D, Abate KH, Abay SM, Abbafati C, Abbasi N, et al. Global, regional, and national incidence, prevalence, and years lived with disability for 354 diseases and injuries for 195 countries and territories, 1990–2017: a systematic analysis for the Global Burden of Disease Study 2017. The Lancet. 2018;392: 1789–1858. doi: 10.1016/S0140-6736(18)32279-7 30496104 PMC6227754

[86] Sunderland EM, Hu XC, Dassuncao C, Tokranov AK, Wagner CC, Allen JG. A review of the pathways of human exposure to poly- and perfluoroalkyl substances (PFASs) and present understanding of health effects. Journal of Exposure Science & Environmental Epidemiology. 2019;29: 131–147. doi: 10.1038/s41370-018-0094-1 30470793 PMC6380916

[87] Granum B, Haug LS, Namork E, Stølevik SB, Thomsen C, Aaberge IS, et al. Pre-natal exposure to perfluoroalkyl substances may be associated with altered vaccine antibody levels and immune-related health outcomes in early childhood. Journal of Immunotoxicology. 2013;10: 373–379. doi: 10.3109/1547691X.2012.755580 23350954

[88] Wright SL, Kelly FJ. Plastic and Human Health: A Micro Issue? Environ Sci Technol. 2017;51: 6634–6647. doi: 10.1021/acs.est.7b00423 28531345

[89] Smith M, Love DC, Rochman CM, Neff RA. Microplastics in Seafood and the Implications for Human Health. Curr Envir Health Rpt. 2018;5: 375–386. doi: 10.1007/s40572-018-0206-z 30116998 PMC6132564

[90] Brown HCP, Smit B, Somorin OA, Sonwa DJ, Ngana F. Institutional perceptions, adaptive capacity and climate change response in a post-conflict country: a case study from Central African Republic. Climate and Development. 2013;5: 206–216.

[91] Milman A, Bunclark L, Conway D, Adger WN. Assessment of institutional capacity to adapt to climate change in transboundary river basins. Climatic Change. 2013;121: 755–770.

[92] Ostrom E. Collective action and the evolution of social norms. Journal of Natural Resources Policy Research. 2014;6: 235–252.

[93] Ostrom E. Polycentric systems for coping with collective action and global environmental change. Global Environmental Change. 2010;20: 550–557. doi: 10.1016/j.gloenvcha.2010.07.004

[94] Ostrom E. Beyond Markets and States: Polycentric Governance of Complex Economic Systems. American Economic Review. 2010;100: 641–672. doi: 10.1257/aer.100.3.641

[95] Adger N, Arnell NW, Tompkins EL. Successful adaptation to climate change across scales. Global Environmental Change. 2005;15: 77–86. doi: 10.1016/j.gloenvcha.2004.12.005

[96] Martinich J, Crimmins A. Climate damages and adaptation potential across diverse sectors of the United States. Nature Climate Change. 2019;9: 397. doi: 10.1038/s41558-019-0444-6 31031825 PMC6483104

[97] Olmstead SM, Stavins RN. Three key elements of a post-2012 international climate policy architecture. Review of Environmental Economics and Policy. 2012;6: 65–85.

[98] Kraft ME. Environmental Policy and Politics. Taylor & Francis; 2017.

[99] Agrawal A. Studying the commons, governing common-pool resource outcomes: Some concluding thoughts. Environmental Science & Policy. 2014;36: 86–91. doi: 10.1016/j.envsci.2013.08.012

[100] Wild CP. Complementing the Genome with an “Exposome”: The Outstanding Challenge of Environmental Exposure Measurement in Molecular Epidemiology. Cancer Epidemiol Biomarkers Prev. 2005;14: 1847–1850. doi: 10.1158/1055-9965.EPI-05-0456 16103423

[101] Moe SJ, Schamphelaere KD, Clements WH, Sorensen MT, Brink PJV den, Liess M. Combined and interactive effects of global climate change and toxicants on populations and communities. Environmental Toxicology and Chemistry. 2013;32: 49–61. doi: 10.1002/etc.2045 23147390 PMC3601420

[102] Teron L, Louis-Charles HM, Nibbs F, Uppalapati SS. Establishing a Toxics Mobility Inventory for Climate Change and Pollution. Sustainability. 2019;12: 226–234. doi: 10.1089/sus.2019.0003

[103] UNDP. Human Development Index (HDI) | Human Development Reports. Geneva, Switzerland: United Nations Development Program; 2019 p. 2. Available: http://hdr.undp.org/en/content/human-development-index-hdi.

[104] Kallis G, Kostakis V, Lange S, Muraca B, Paulson S, Schmelzer M. Research On Degrowth. Annual Review of Environment and Resources. 2018;43: 291–316. doi: 10.1146/annurev-environ-102017-025941

[105] Martinez-Alier J. The environmentalism of the poor. Geoforum. 2014;54: 239–241. doi: 10.1016/j.geoforum.2013.04.019

[106] Hickel J. Quantifying national responsibility for climate breakdown: an equality-based attribution approach for carbon dioxide emissions in excess of the planetary boundary. The Lancet Planetary Health. 2020;4: e399–e404. doi: 10.1016/S2542-5196(20)30196-0 32918885

[107] Hickel J. Is it possible to achieve a good life for all within planetary boundaries? Third World Quarterly. 2019;40: 18–35. doi: 10.1080/01436597.2018.1535895

[108] Adger WN, de Campos RS, Siddiqui T, Gavonel MF, Szaboova L, Rocky MH, et al. Human security of urban migrant populations affected by length of residence and environmental hazards. Journal of Peace Research. 2021;58: 50–66. doi: 10.1177/0022343320973717

[109] Perera FP. Multiple Threats to Child Health from Fossil Fuel Combustion: Impacts of Air Pollution and Climate Change. Environ Health Perspect. 2017;125: 141–148. doi: 10.1289/EHP299 27323709 PMC5289912

[110] Noyes PD, McElwee MK, Miller HD, Clark BW, Van Tiem LA, Walcott KC, et al. The toxicology of climate change: Environmental contaminants in a warming world. Environment International. 2009;35: 971–986. doi: 10.1016/j.envint.2009.02.006 19375165

[111] World Bank. Natural Hazards, UnNatural Disasters: the economics of effective prevention. Washington, D.C.: World Bank; 2010.

[112] Temper L, Bene D del, Martinez-Alier J. Mapping the frontiers and front lines of global environmental justice: the EJAtlas. Journal of Political Ecology. 2015;22: 255–278. doi: 10.2458/v22i1.21108

[113] Nkanga MSN, Longo-Mbenza B, Adeniyi OV, Ngwidiwo JB, Katawandja AL, Kazadi PRB, et al. Ageing, exposure to pollution, and interactions between climate change and local seasons as oxidant conditions predicting incident hematologic malignancy at KINSHASA University clinics, Democratic Republic of CONGO (DRC). BMC Cancer. 2017;17: 559. doi: 10.1186/s12885-017-3547-3 28835214 PMC5569529

[114] Lateef ASA, Fernandez-Alonso M, Tack L, Delvaux D. Geological constraints on urban sustainability, Kinshasa City, Democratic Republic of Congo. Environmental Geosciences. 2010;17: 17–35. doi: 10.1306/eg.04080908007

[115] Kabamba M, Basosila N, Mulaji C, Mata H, Tuakuila J. Toxic Heavy Metals in Ambient Air of Kinshasa, Democratic Republic Congo. J Environ Anal Chem. 2016;3: 1–4. doi: 10.4172/2380-2391.1000178

[116] Hales S, Kovats S, Lloyd S, Campbell-Lendrum D. Quantitative risk assessment of the effects of climate change on selected causes of death, 2030s and 2050s. Geneva, Switzerland: World Health Orgnaization; 2014 p. 128.

[117] Han L, Sun Z, Gong T, Zhang X, He J, Xing Q, et al. Assessment of the short-term mortality effect of the national action plan on air pollution in Beijing, China. Environ Res Lett. 2020;15: 034052. doi: 10.1088/1748-9326/ab6f13

[118] Chen K, Wang M, Huang C, Kinney PL, Anastas PT. Air pollution reduction and mortality benefit during the COVID-19 outbreak in China. The Lancet Planetary Health. 2020;4: e210–e212. doi: 10.1016/S2542-5196(20)30107-8 32411944 PMC7220178

[119] Quéré CL, Jackson RB, Jones MW, Smith AJP, Abernethy S, Andrew RM, et al. Temporary reduction in daily global CO 2 emissions during the COVID-19 forced confinement. Nat Clim Chang. 2020; 1–7. doi: 10.1038/s41558-020-0797-x

[120] Acuto M. COVID-19: Lessons for an Urban(izing) World. One Earth. 2020;2: 317–319. doi: 10.1016/j.oneear.2020.04.004 34171028 PMC7159854

[121] Hsiang S, Allen D, Annan-Phan S, Bell K, Bolliger I, Chong T, et al. The effect of large-scale anti-contagion policies on the COVID-19 pandemic. Nature. 2020; 1–9. doi: 10.1038/s41586-020-2404-8 32512578

[122] Steffen W, Persson Å, Deutsch L, Zalasiewicz J, Williams M, Richardson K, et al. The Anthropocene: From Global Change to Planetary Stewardship. AMBIO. 2011;40: 739–761. doi: 10.1007/s13280-011-0185-x 22338713 PMC3357752

