# Supplementary figures and images for: Global distribution and coincidence of pollution, climate impacts, and health risk in the Anthropocene
Source: PLoS One. 2021 Jul 21;16(7):e0254060. doi: 10.1371/journal.pone.0254060 (PMC8294505; doi:10.1371/journal.pone.0254060)

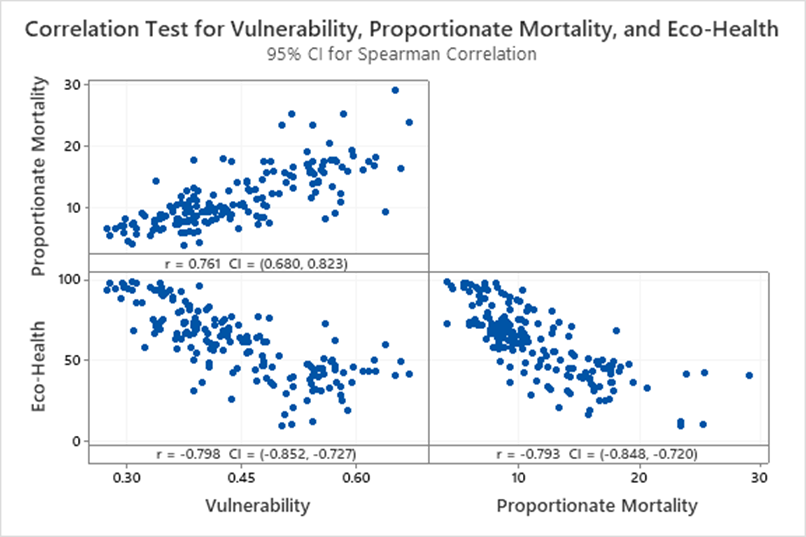

Supplement: S1 Fig — (TIF) [file pone.0254060.s001.tif]
